# *N*′-(2-Meth­oxy-1-naphthyl­idene)nicotino­hydrazide

**DOI:** 10.1107/S1600536810003843

**Published:** 2010-02-06

**Authors:** Cong Li, Ping Wang, Yong-Qing Su

**Affiliations:** a Faculty of Chemistry and Chemical Engineering, Yunnan Normal University, Kunming 650092, People’s Republic of China

## Abstract

The title compound, C_18_H_15_N_3_O_2_, was prepared by the reaction of 2-methoxy­naphthaldehyde with nicotinic acid hydrazide in methanol. The dihedral angle between the naphthalene ring system and the pyridine ring is 9.2 (3)°. An intra­molecular C—H⋯N hydrogen bond is observed. In the crystal structure, mol­ecules are linked into chains running along the *c* axis by inter­molecular N—H⋯O hydrogen bonds.

## Related literature

For general background to Schiff base compounds, see: Archibald *et al.* (1994 ▶); Harada *et al.* (1999 ▶); Ogawa *et al.* (1998 ▶). For related structures, see: Mohd Lair *et al.* (2009 ▶); Sun *et al.* (2009 ▶); Wen *et al.* (2009 ▶).
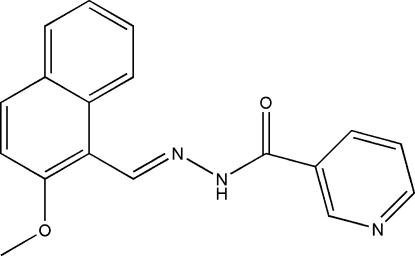


## Experimental

### 

#### Crystal data

C_18_H_15_N_3_O_2_
*M* *_r_* = 305.33
Tetragonal, 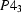

*a* = 9.6163 (13) Å
*c* = 17.442 (3) Å
*V* = 1612.9 (4) Å^3^
*Z* = 4
Mo *K*α radiation
μ = 0.08 mm^−1^
*T* = 298 K
0.23 × 0.21 × 0.21 mm

#### Data collection

Bruker APEXII CCD area-detector diffractometer
Absorption correction: multi-scan (*SADABS*; Sheldrick, 2004 ▶) *T*_min_ = 0.981, *T*_max_ = 0.983
8478 measured reflections
1498 independent reflections
1109 reflections with *I* > 2σ(*I*)
*R*_int_ = 0.040

#### Refinement

*R*[*F* ^2^ > 2σ(*F* ^2^)] = 0.038
*wR*(*F* ^2^) = 0.099
*S* = 1.03
1498 reflections
212 parameters
2 restraints
H atoms treated by a mixture of independent and constrained refinement
Δρ_max_ = 0.10 e Å^−3^
Δρ_min_ = −0.11 e Å^−3^

### 

Data collection: *APEX2* (Bruker, 2004 ▶); cell refinement: *SAINT* (Bruker, 2004 ▶); data reduction: *SAINT*; program(s) used to solve structure: *SHELXS97* (Sheldrick, 2008 ▶); program(s) used to refine structure: *SHELXL97* (Sheldrick, 2008 ▶); molecular graphics: *SHELXTL* (Sheldrick, 2008 ▶); software used to prepare material for publication: *SHELXTL*.

## Supplementary Material

Crystal structure: contains datablocks global, I. DOI: 10.1107/S1600536810003843/ci5027sup1.cif

Structure factors: contains datablocks I. DOI: 10.1107/S1600536810003843/ci5027Isup2.hkl

Additional supplementary materials:  crystallographic information; 3D view; checkCIF report

## Figures and Tables

**Table 1:** Hydrogen-bond geometry (Å, °)

*D*—H⋯*A*	*D*—H	H⋯*A*	*D*⋯*A*	*D*—H⋯*A*
N2—H2⋯O2^i^	0.90 (1)	2.00 (2)	2.833 (3)	154 (3)
C9—H9⋯N1	0.93	2.30	2.930 (5)	125

Symmetry code: (i) 
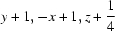
.

**Fig. 1.**
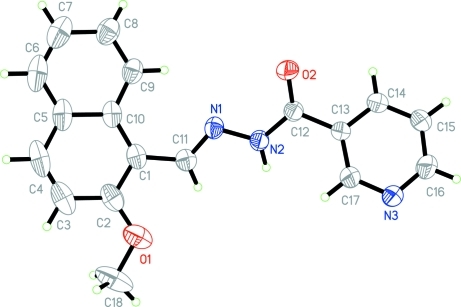
The molecular structure of the title compound, showing the atom-numbering scheme. Displacement ellipsoids are drawn at the 30% probability level.

**Fig. 2.**
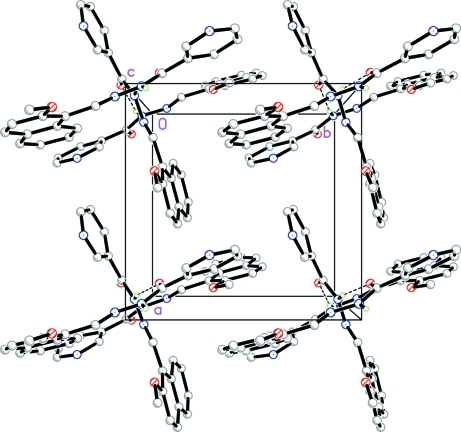
The crystal packing of the title compound. Intermolecular hydrogen bonds are shown as dashed lines.

## supplementary crystallographic information

### Comment

Schiff bases have been received much attention in recent years (Ogawa *et al.*, 1998; Archibald *et al.*, 1994; Harada *et al.*, 1999). As a further investigation of the structures of Schiff base compounds, the title new compound is reported here.

In the title compound, the dihedral angle between the naphthalene ring system and the pyridine ring is 9.2 (3)°. The bond lengths are comparable to those observed in related Schiff base compounds (Wen *et al.*, 2009; Mohd Lair *et al.*, 2009; Sun *et al.*, 2009).

In the crystal structure, molecules form chains running along the *c* axis through intermolecular N—H···O hydrogen bonds (Table 1 and Fig. 2).

### Experimental

2-Methoxynaphthaldehyde (1.0 mmol, 186 mg) and nicotinic acid hydrazide (1.0 mmol, 137 mg) were dissolved in methanol (30 ml). The mixture was stirred at room temperature for 1 h to give a colourless solution. After keeping the solution in air for 5 d, colourless block shaped crystals were formed.

### Refinement

Atom H2 was located in a difference Fourier map and refined isotropically, with the N–H distance restrained to 0.90 (1) Å. The other H atoms were placed in idealized positions and constrained to ride on their parent atoms, with C–H distances in the range 0.93-0.96 Å, and with U_iso_(H) = 1.2 or 1.5U_eq_(C). In the absence of significant anomalous dispersion effects, Friedel pairs were merged before the final refinement.

### Crystal data

**Table:**

C_18_H_15_N_3_O_2_	*D*_x_ = 1.257 Mg m^−^^3^
*M**_r_* = 305.33	Mo *K*α radiation, λ = 0.71073 Å
Tetragonal, *P*4_3_	Cell parameters from 1467 reflections
Hall symbol: P 4cw	θ = 2.4–24.5°
*a* = 9.6163 (13) Å	µ = 0.08 mm^−^^1^
*c* = 17.442 (3) Å	*T* = 298 K
*V* = 1612.9 (4) Å^3^	Block, colourless
*Z* = 4	0.23 × 0.21 × 0.21 mm
*F*(000) = 640	

### Data collection

**Table:**

Bruker APEXII CCD area-detector diffractometer	1498 independent reflections
Radiation source: fine-focus sealed tube	1109 reflections with *I* > 2σ(*I*)
graphite	*R*_int_ = 0.040
ω scans	θ_max_ = 25.2°, θ_min_ = 2.1°
Absorption correction: multi-scan (*SADABS*; Sheldrick, 2004)	*h* = −11→11
*T*_min_ = 0.981, *T*_max_ = 0.983	*k* = −11→8
8478 measured reflections	*l* = −20→20

### Refinement

**Table:**

Refinement on *F*^2^	Primary atom site location: structure-invariant direct methods
Least-squares matrix: full	Secondary atom site location: difference Fourier map
*R*[*F*^2^ > 2σ(*F*^2^)] = 0.038	Hydrogen site location: inferred from neighbouring sites
*wR*(*F*^2^) = 0.099	H atoms treated by a mixture of independent and constrained refinement
*S* = 1.03	*w* = 1/[σ^2^(*F*_o_^2^) + (0.054*P*)^2^ + 0.0224*P*] where *P* = (*F*_o_^2^ + 2*F*_c_^2^)/3
1498 reflections	(Δ/σ)_max_ = 0.001
212 parameters	Δρ_max_ = 0.10 e Å^−^^3^
2 restraints	Δρ_min_ = −0.11 e Å^−^^3^

### Special details

**Table:**

Geometry. All esds (except the esd in the dihedral angle between two l.s. planes) are estimated using the full covariance matrix. The cell esds are taken into account individually in the estimation of esds in distances, angles and torsion angles; correlations between esds in cell parameters are only used when they are defined by crystal symmetry. An approximate (isotropic) treatment of cell esds is used for estimating esds involving l.s. planes.
Refinement. Refinement of *F*^2^ against ALL reflections. The weighted *R*-factor wR and goodness of fit *S* are based on *F*^2^, conventional *R*-factors *R* are based on *F*, with *F* set to zero for negative *F*^2^. The threshold expression of *F*^2^ > σ(*F*^2^) is used only for calculating *R*-factors(gt) etc. and is not relevant to the choice of reflections for refinement. *R*-factors based on *F*^2^ are statistically about twice as large as those based on *F*, and *R*- factors based on ALL data will be even larger.

### Fractional atomic coordinates and isotropic or equivalent isotropic displacement parameters (Å^2^)

**Table:**

	*x*	*y*	*z*	*U*_iso_*/*U*_eq_	
N1	0.9936 (3)	0.0960 (3)	0.18164 (15)	0.0629 (7)	
N2	1.0362 (3)	−0.0311 (3)	0.20995 (14)	0.0624 (7)	
N3	1.2654 (4)	−0.3482 (3)	0.31458 (18)	0.1112 (13)	
O1	0.9212 (3)	0.3474 (3)	0.35200 (18)	0.1066 (9)	
O2	1.1195 (2)	−0.1023 (2)	0.09477 (13)	0.0791 (7)	
C1	0.8973 (3)	0.3191 (3)	0.2190 (2)	0.0670 (9)	
C2	0.8904 (4)	0.4054 (4)	0.2829 (3)	0.0843 (11)	
C3	0.8547 (5)	0.5467 (5)	0.2750 (4)	0.1166 (17)	
H3	0.8516	0.6039	0.3179	0.140*	
C4	0.8256 (5)	0.5980 (5)	0.2064 (5)	0.1223 (19)	
H4	0.8060	0.6924	0.2022	0.147*	
C5	0.8231 (4)	0.5148 (4)	0.1387 (3)	0.0943 (12)	
C6	0.7859 (5)	0.5685 (5)	0.0671 (4)	0.1232 (19)	
H6	0.7606	0.6616	0.0632	0.148*	
C7	0.7857 (5)	0.4884 (6)	0.0029 (4)	0.1166 (16)	
H7	0.7618	0.5270	−0.0441	0.140*	
C8	0.8218 (4)	0.3472 (5)	0.0079 (3)	0.0914 (12)	
H8	0.8211	0.2915	−0.0357	0.110*	
C9	0.8579 (3)	0.2925 (4)	0.0773 (2)	0.0750 (9)	
H9	0.8816	0.1988	0.0799	0.090*	
C10	0.8609 (3)	0.3720 (3)	0.1454 (2)	0.0691 (9)	
C11	0.9425 (3)	0.1769 (3)	0.2331 (2)	0.0653 (8)	
H11	0.9337	0.1422	0.2826	0.078*	
C12	1.1047 (3)	−0.1204 (3)	0.16398 (18)	0.0585 (8)	
C13	1.1660 (3)	−0.2433 (3)	0.20306 (16)	0.0588 (8)	
C14	1.1928 (4)	−0.3620 (4)	0.1619 (2)	0.0806 (10)	
H14	1.1688	−0.3673	0.1104	0.097*	
C15	1.2545 (5)	−0.4718 (4)	0.1972 (2)	0.1045 (14)	
H15	1.2742	−0.5528	0.1703	0.125*	
C16	1.2870 (6)	−0.4605 (4)	0.2729 (3)	0.1184 (17)	
H16	1.3270	−0.5371	0.2968	0.142*	
C17	1.2047 (4)	−0.2419 (4)	0.2787 (2)	0.0835 (11)	
H17	1.1875	−0.1616	0.3069	0.100*	
C18	0.8999 (7)	0.4279 (7)	0.4200 (3)	0.161 (2)	
H18A	0.9667	0.5021	0.4218	0.241*	
H18B	0.9112	0.3695	0.4642	0.241*	
H18C	0.8077	0.4661	0.4197	0.241*	
H2	1.019 (4)	−0.056 (3)	0.2588 (9)	0.080*	

### Atomic displacement parameters (Å^2^)

**Table:**

	*U*^11^	*U*^22^	*U*^33^	*U*^12^	*U*^13^	*U*^23^
N1	0.0601 (15)	0.0596 (15)	0.0689 (16)	0.0083 (12)	0.0102 (12)	0.0094 (13)
N2	0.0667 (17)	0.0605 (15)	0.0601 (16)	0.0105 (12)	0.0121 (13)	0.0075 (13)
N3	0.178 (3)	0.082 (2)	0.074 (2)	0.049 (2)	−0.040 (2)	−0.0153 (17)
O1	0.104 (2)	0.120 (2)	0.096 (2)	−0.0024 (16)	−0.0023 (16)	−0.038 (2)
O2	0.0954 (16)	0.0891 (16)	0.0528 (14)	0.0243 (12)	0.0153 (12)	0.0115 (11)
C1	0.0482 (17)	0.0588 (19)	0.094 (3)	−0.0012 (13)	0.0115 (16)	−0.0046 (19)
C2	0.064 (2)	0.073 (2)	0.117 (4)	−0.0071 (18)	0.009 (2)	−0.025 (3)
C3	0.113 (4)	0.073 (3)	0.163 (5)	−0.006 (2)	0.015 (4)	−0.035 (3)
C4	0.113 (4)	0.054 (2)	0.200 (6)	0.001 (2)	0.022 (4)	−0.020 (4)
C5	0.078 (2)	0.058 (2)	0.147 (4)	0.0043 (18)	0.015 (3)	0.014 (3)
C6	0.106 (3)	0.071 (3)	0.193 (6)	0.017 (2)	0.009 (4)	0.039 (4)
C7	0.096 (3)	0.110 (4)	0.143 (5)	0.011 (3)	0.002 (3)	0.049 (4)
C8	0.074 (2)	0.096 (3)	0.105 (3)	0.011 (2)	−0.001 (2)	0.021 (2)
C9	0.0583 (19)	0.073 (2)	0.094 (3)	0.0055 (16)	0.0041 (18)	0.014 (2)
C10	0.0476 (16)	0.0550 (18)	0.105 (3)	−0.0014 (14)	0.0118 (17)	0.0053 (19)
C11	0.0597 (18)	0.0667 (19)	0.070 (2)	−0.0011 (15)	0.0076 (16)	0.0000 (17)
C12	0.0563 (18)	0.0623 (19)	0.057 (2)	0.0047 (13)	0.0063 (15)	0.0040 (15)
C13	0.0664 (19)	0.0583 (18)	0.0516 (18)	0.0058 (13)	0.0016 (14)	−0.0006 (14)
C14	0.110 (3)	0.073 (2)	0.059 (2)	0.0182 (19)	0.0007 (19)	−0.0036 (18)
C15	0.164 (4)	0.075 (3)	0.075 (3)	0.039 (2)	−0.014 (3)	−0.018 (2)
C16	0.189 (5)	0.075 (3)	0.091 (3)	0.048 (3)	−0.035 (3)	−0.002 (2)
C17	0.110 (3)	0.069 (2)	0.071 (3)	0.0228 (19)	−0.022 (2)	−0.0121 (18)
C18	0.171 (5)	0.181 (5)	0.130 (5)	−0.031 (4)	−0.007 (4)	−0.088 (4)

### Geometric parameters (Å, °)

**Table:**

N1—C11	1.285 (4)	C6—H6	0.93
N1—N2	1.381 (3)	C7—C8	1.404 (7)
N2—C12	1.347 (4)	C7—H7	0.93
N2—H2	0.899 (10)	C8—C9	1.365 (5)
N3—C16	1.318 (5)	C8—H8	0.93
N3—C17	1.333 (4)	C9—C10	1.413 (5)
O1—C2	1.361 (5)	C9—H9	0.93
O1—C18	1.431 (6)	C11—H11	0.93
O2—C12	1.228 (4)	C12—C13	1.486 (4)
C1—C2	1.391 (5)	C13—C17	1.371 (4)
C1—C10	1.425 (5)	C13—C14	1.373 (4)
C1—C11	1.455 (4)	C14—C15	1.358 (5)
C2—C3	1.408 (6)	C14—H14	0.93
C3—C4	1.324 (7)	C15—C16	1.361 (6)
C3—H3	0.93	C15—H15	0.93
C4—C5	1.427 (7)	C16—H16	0.93
C4—H4	0.93	C17—H17	0.93
C5—C6	1.398 (8)	C18—H18A	0.96
C5—C10	1.425 (5)	C18—H18B	0.96
C6—C7	1.358 (7)	C18—H18C	0.96
			
C11—N1—N2	113.5 (3)	C10—C9—H9	118.6
C12—N2—N1	119.8 (2)	C9—C10—C1	124.7 (3)
C12—N2—H2	119 (2)	C9—C10—C5	116.5 (4)
N1—N2—H2	121 (2)	C1—C10—C5	118.8 (4)
C16—N3—C17	116.0 (3)	N1—C11—C1	124.4 (3)
C2—O1—C18	118.8 (4)	N1—C11—H11	117.8
C2—C1—C10	119.8 (3)	C1—C11—H11	117.8
C2—C1—C11	116.1 (3)	O2—C12—N2	123.5 (3)
C10—C1—C11	124.1 (3)	O2—C12—C13	121.2 (3)
O1—C2—C1	117.0 (3)	N2—C12—C13	115.3 (3)
O1—C2—C3	122.3 (5)	C17—C13—C14	117.4 (3)
C1—C2—C3	120.6 (5)	C17—C13—C12	122.8 (3)
C4—C3—C2	120.0 (5)	C14—C13—C12	119.7 (3)
C4—C3—H3	120.0	C15—C14—C13	119.4 (3)
C2—C3—H3	120.0	C15—C14—H14	120.3
C3—C4—C5	122.9 (4)	C13—C14—H14	120.3
C3—C4—H4	118.6	C14—C15—C16	118.5 (3)
C5—C4—H4	118.6	C14—C15—H15	120.7
C6—C5—C10	119.7 (5)	C16—C15—H15	120.7
C6—C5—C4	122.5 (5)	N3—C16—C15	124.3 (4)
C10—C5—C4	117.9 (5)	N3—C16—H16	117.8
C7—C6—C5	121.8 (4)	C15—C16—H16	117.8
C7—C6—H6	119.1	N3—C17—C13	124.3 (3)
C5—C6—H6	119.1	N3—C17—H17	117.9
C6—C7—C8	119.8 (5)	C13—C17—H17	117.9
C6—C7—H7	120.1	O1—C18—H18A	109.5
C8—C7—H7	120.1	O1—C18—H18B	109.5
C9—C8—C7	119.3 (5)	H18A—C18—H18B	109.5
C9—C8—H8	120.3	O1—C18—H18C	109.5
C7—C8—H8	120.3	H18A—C18—H18C	109.5
C8—C9—C10	122.8 (4)	H18B—C18—H18C	109.5
C8—C9—H9	118.6		

### Hydrogen-bond geometry (Å, °)

**Table:**

*D*—H···*A*	*D*—H	H···*A*	*D*···*A*	*D*—H···*A*
N2—H2···O2^i^	0.90 (1)	2.00 (2)	2.833 (3)	154 (3)
C9—H9···N1	0.93	2.30	2.930 (5)	125

Symmetry codes: (i) *y*+1, −*x*+1, *z*+1/4.
